# Mosaic DNA Imports with Interspersions of Recipient Sequence after Natural Transformation of *Helicobacter pylori*

**DOI:** 10.1371/journal.pone.0003797

**Published:** 2008-11-24

**Authors:** Stefan Kulick, Claudia Moccia, Xavier Didelot, Daniel Falush, Christian Kraft, Sebastian Suerbaum

**Affiliations:** 1 Institute of Medical Microbiology and Hospital Epidemiology, Hannover Medical School, Hannover, Germany; 2 Department of Statistics, University of Warwick, Coventry, United Kingdom; 3 Environmental Research Institute, University College Cork, Cork, Ireland; Centre for DNA Fingerprinting and Diagnostics, India

## Abstract

*Helicobacter pylori* colonizes the gastric mucosa of half of the human population, causing gastritis, ulcers, and cancer. *H. pylori* is naturally competent for transformation by exogenous DNA, and recombination during mixed infections of one stomach with multiple *H. pylori* strains generates extensive allelic diversity. We developed an *in vitro* transformation protocol to study genomic imports after natural transformation of *H. pylori*. The mean length of imported fragments was dependent on the combination of donor and recipient strain and varied between 1294 bp and 3853 bp. In about 10% of recombinant clones, the imported fragments of donor DNA were interrupted by short interspersed sequences of the recipient (ISR) with a mean length of 82 bp. 18 candidate genes were inactivated in order to identify genes involved in the control of import length and generation of ISR. Inactivation of the antimutator glycosylase MutY increased the length of imports, but did not have a significant effect on ISR frequency. Overexpression of *mutY* strongly increased the frequency of ISR, indicating that MutY, while not indispensable for ISR formation, is part of at least one ISR-generating pathway. The formation of ISR in *H. pylori* increases allelic diversity, and contributes to the uniquely low linkage disequilibrium characteristic of this pathogen.

## Introduction

*Helicobacter pylori* infects an estimated 50% of the world population, and can result in chronic gastritis, gastric or duodenal ulcers, gastric cancer, and MALT lymphoma [1]. Among the most striking characteristics of *H. pylori* are its amazing allelic diversity and variability. Not only do almost all infected persons harbor their own unique strain(s), but *H. pylori* can undergo genetic change during chronic colonization. Such change is driven by the combination of frequent recombination between strains that simultaneously colonize one stomach, and an elevated mutation rate that is thought to be at least partly due to the lack of a MutHLS mismatch repair pathway (*H. pylori* possesses a MutS homolog, which, however, is not involved in mismatch repair [2], for reviews, see [3], [4]).

*H. pylori* is naturally competent, and takes up exogenous DNA through an unusual transport system related to type IV secretion systems [5], [6]. Population genetic analysis of *H. pylori* nucleotide sequences has provided strong evidence that recombination is far more common in *H. pylori* than in other bacteria [7], [8]. More direct information about the effects of recombination on *H. pylori* comes from studies of sequential *H. pylori* isolates, cultured from biopsies taken from the same patient at time intervals of several months to years [9]. Numerous recombination events, many of them spanning only a few hundred base pairs, were detected when 10 gene fragments were sequenced for 24 pairs of such sequential isolates. Bayesian analysis revealed the most likely combination of recombination rate, mutation rate, and length of imported fragments that would generate the dataset. Strikingly, *H. pylori* cells that undergo recombination import unusually short pieces of DNA into their genomes (estimated mean of 417 bp), in contrast to other bacteria, where known lengths range from 2 kb (pneumococci, ref. [10]) to over 10 kb (*E. coli*, *Bacillus subtilis*, refs. [11], [12]). However, despite ample evidence of the importance of recombination to the population structure of *H. pylori* [7], [13], [14], few studies have addressed basic questions of the recombination process. We have developed an *in vitro* transformation protocol that permits to study import events after natural transformation and to assess the role of candidate genes in determining import frequency and length. The data show that import of short fragments of DNA into the chromosome is an intrinsic property of the *H. pylori* DNA recombination/repair machinery. Imports can be interrupted by interspersed sequences of the recipient (ISR) of varying length which can accelerate allelic diversification. Overexpression of the DNA glycosylase MutY increased the frequency of ISR, suggesting that MutY activity is involved in at least one pathway leading to the formation of ISR.

## Results

### Short sequence imports after natural transformation of H. pylori

**Figure 1:**
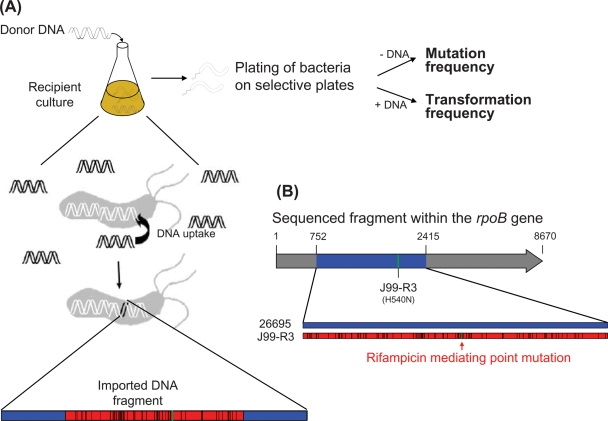
Schematic of the *H. pylori in vitro* transformation system used to study import events after natural transformation. (A) In the transformation experiment, *H. pylori* recipient strains were incubated with genomic DNA of a Rif resistant *H. pylori* donor strain. Bacteria were spread on selective plates and counted to determine the frequencies of resistant clones (transformants plus spontaneous mutants). Mutation frequencies were determined by plating similarly treated bacteria without addition of DNA. (B) To calculate the DNA import lengths, a 1663 bp long fragment (blue) of *rpoB* including the Rif resistant mediating point mutation of the donor strain was sequenced and compared to the sequences of the recipient and the donor (here: 26695 [blue] and J99-R3 [red]). The *rpoB* alleles of recipient and donor differ by 3.7–5.5% of nucleotides, depending on the strain combination used. Sequence comparisons of the recombinant sequence with both donor and recipient *rpoB* sequence permit to determine approximate starting or/and end points of DNA imports.

In order to study the characteristics of import events after natural transformation of *H. pylori*, we transformed Rif sensitive *H. pylori* strains 26695, N6, or J99 with genomic DNA purified from Rif resistant *H. pylori* donor strains, J99-R3, 26695-R1, and N6-R1, and selected Rif resistant clones from these transformation experiments. In a parallel experiment without addition of DNA, the frequency of spontaneous mutations to Rif resistance was determined (Fig. 1A). The spontaneous mutation frequencies were 1.1×10^−6^ (26695), 1.6×10^−6^ (J99), and 4.6×10^−7^ (N6), consistent with the results of earlier studies [15], [16]. Addition of DNA from a Rif resistant donor strain to liquid cultures increased the frequency of Rif resistant clones by a factor of 22–199 depending on the recipient/donor combination, due to natural transformation (Table S4).

**Figure 2:**
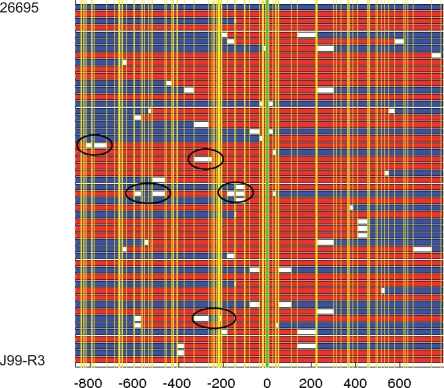
Import events after transformation of recipient strain 26695 with DNA of Rif resistant strain J99-R3. Each row represents a 1663 bp partial *rpoB* sequence. The blue row on top represents the sequence of the recipient strain 26695, and the red row at the bottom that of the donor strain J99-R3. Vertical yellow lines represent the positions of polymorphic sites, the green line depicts the position of the point mutation that is responsible for Rif resistance in J99-R3. Numbers below the panel: position relative to the Rif resistance point mutation, negative values indicate upstream nucleotides. The fifty rows between 26695 and J99-R3 depict the sequences of 50 randomly selected clones out of 95 recombinant clones analyzed. Any fragment surrounded by two sites identical to the donor is shown in red, any fragment surrounded by two sites identical to the recipient is shown in blue, and the remainder of the sequence is in white. Consequently, each sequence is shown as a mosaic of colors, where blue indicates DNA from the recipient, red DNA from the donor, and white DNA of unresolved origin. ISR are surrounded by circles. Note that ISR containing only one recipient-specific polymorphism will be represented as a white box separated by a vertical line indicating the polymorphism.

Rif resistant clones were characterized by nucleotide sequence analysis of a 1663 bp fragment of *rpoB* that included the positions conferring Rif resistance in the donors (Fig. 1B). The *rpoB* fragments of the three wild type strains differed at 3.7–5.5% of nucleotides. Due to this high degree of sequence divergence, even small imports created a mosaic sequence, and in many cases, both start and end point of DNA import events could be mapped with high accuracy by comparing the mosaic recombinant sequence with both donor and recipient sequences (Fig. 2). In other cases, the import extended beyond the sequenced fragment, providing only a minimum bound for the length of the import. We therefore applied a Bayesian model to the dataset in order to estimate the mean length of imports. Our estimates varied between 1294 bp (J99/26695-R1) and 3853 bp (N6/J99-R3), and the differences observed between some recipient/donor combinations were statistically significant (Table 1). The length of import events was thus longer than previously observed in sequential patient isolates (417 bp, ref. [9]), but still shorter than values reported for transformation in most other bacterial species.

**Table 1:** Maximum likelihood estimation (MLE) of the mean length of donor DNA imports in the *rpoB* gene, number of clones with ISR in the imported DNA fragments and MLE of ISR length of *H. pylori* wild type strains.

Recipient	Donor	Num clones^1^	MLE of import length (bp)	Bayes Factor^2^	Clones with ISR	Bayes Factor^2^	MLE of ISR (bp)	Bayes Factor^2^
**26695**	**J99-R3**	95	1681		8		39	
	**N6-R1**	26	2434	0.29	3	0.19	121	1.20
**J99**	**26695-R1**	32	1294	0.19	4	0.20	38	0.24
**N6**	**26695-R1**	25	3819*	28.21	5	0.66	110	1.62
	**J99-R3**	80	3853*	4.43×10^4^	8	0.12	114	2.26

See Methods for interpretation of Bayes Factor values. Strongly significant results (Bayes Factor ≥10) are marked with an asterisk.

1 Number of clones with DNA imports in *rpoB.*

2 Approximated using the Bayesian Information Criterion (cf. Methods).

### Interspersed sequences of the recipient (ISR) within imports

When the *rpoB* sequences of Rif resistant transformants were compared with those of corresponding donor and recipient, we found that for ∼10% of the recombinant clones, the stretch of integrated DNA from the Rif resistant donor was interrupted by one or multiple short fragments where the sequence was identical to that of the recipient. These interruptions of the imported fragment were termed ‘interspersed sequences of the recipient’ (ISR, Fig. 2). Some ISR included only a single polymorphic nucleotide, while others were up to 593 bp long, including up to 21 recipient-specific nucleotides. ISR could be observed in 8 out of 95 clones with DNA import in the recipient/donor combination 26695/J99-R3, 3 out of 26 in 26695/N6-R1, 4 out of 32 in J99/26695-R1, 5 out of 25 in N6/26695-R1, and 8 out of 80 clones in N6/J99-R3 (Table 1). The estimated mean length of ISR for the wild type strains varied between 38 and 121 bp (Table 1 and S7). However, there was no significant difference of ISR lengths between the analyzed recipient/donor combinations.

### Short import length and ISR do not depend on DNA uptake via the ComB system

We hypothesized that the short length of imported fragments and/or the generation of ISR might be a consequence of the passage of exogenous DNA via the *H. pylori*-specific ComB DNA uptake system. To test this hypothesis, a *comB10* mutant of *H. pylori* 26695 was constructed. After verification that this strain was unable to take up DNA by natural transformation, electroporation was used to introduce J99-R3 DNA into the *comB10* mutant strain. 25 Rif resistant clones with DNA import were generated by this approach. The mean length of imports was not significantly different from the wild type, and 8 clones contained ISR, indicating that DNA transfer through the ComB system was not responsible for either the short length of imports, or the formation of ISR (Table 2). The frequency of ISR (8/25) was significantly higher in clones obtained by electroporation of the *comB10* mutant than in clones isolated after natural transformation of 26695 wild type (8/95; Table 2).

**Table 2:** Import length and ISR formation are independent of the DNA uptake system ComB.

Recipient	Num clones^1^	MLE of import length (bp)	Bayes Factor^2^	Clones with ISR	Bayes Factor^2^
**26695**	95	1681		8	
**26695*comB10***	0	_	_	0	1.00
**26695*comB10* EP^3^**	25	2228	0.15	8*	11.45

J99-R3 DNA was used as donor DNA for all transformations. See Methods for interpretation of Bayes Factor values. Strongly significant results (Bayes Factor ≥10) are marked with an asterisk.

1 Number of analyzed clones with DNA imports.

2 Approximated using the Bayesian Information Criterion (cf. Methods).

3 EP: electroporation.

### Search for genes determining import length and ISR formation

The results from our *in vitro* transformation experiments suggested that importing short pieces of donor DNA into the chromosome was an inherent property of the *H. pylori* DNA recombination or DNA repair machinery. In order to identify genes involved in determining import length and ISR formation, we selected 18 genes with predicted roles in DNA repair or recombination (*magIII*, *mfd*, *mutS*, *mutY*, *nth*, *nucT*, *recA*, *recB*, *recG*, *recJ*, *recN*, *recR*, *ruvABC*, *ung*, *xseA*, and *xth*) and inactivated these genes in *H. pylori* 26695 by allelic disruption. Subsequently, all mutant strains were individually transformed with DNA from strain J99-R3. Mutation and recombination frequencies were measured, and the length of imported fragments and the frequency of ISR were determined by sequence analysis of *rpoB* in Rif resistant clones. Inactivation of *mutY*, *nucT*, *nth*, and *ung* significantly increased the rate of spontaneous mutations to Rif resistance, while disruption of *recA*, *recB*, *ruvA*, *ruvB*, and *ruvC* had the opposite effect. Inactivation of *recA*, *recB*, *recN*, *ruvA*, *ruvB*, and *ruvC* significantly reduced the number of recombinant clones, while disruption of *nth*, *recG*, *recR*, and *xseA* increased it (Table S4). However, out of 18 genes investigated, only one, *mutY*, had a significant effect on import length and ISR formation.

### Role of the DNA glycosylase *mutY* in import length and ISR formation

The *H. pylori* antimutator gene *mutY* (HP0142) encodes a DNA glycosylase involved in preventing specific C to A transversions [16]–[19]. Due to this antimutator function of *mutY*, a 26695 *mutY* mutant had a strongly increased frequency of spontaneous mutation to Rif resistance. In order to test the impact of an inactivation of *mutY* on length of import and ISR, a prescreening procedure was applied to select recombinants from the Rif resistant clones isolated from the transformation experiment. 43 clones with import were selected from a total of 250 colonies screened, and characterized by *rpoB* sequence analysis.

The mean length estimate of import events in the *mutY* mutant was 3268 bp, almost twice of that observed in wild type bacteria. This difference was statistically significant (Table 3). ISR were detected in 10/43 (23%) of recombinant clones; this ISR frequency was, however, not significantly different from wild type.

**Table 3:** Import length and clones with ISR in selected mutants of *H. pylori* 26695 transformed with DNA from J99-R3.

Recipient	Num clones	MLE of import length (bp)	Bayes Factor^1^	Clones with ISR	Bayes Factor
**26695**	95	1681		8	
**26695*magIII***	51	1729	0.06	9	0.53
**26695*mutY***	43	3268*	38.88	9	1.15
**26695*mutY* comp**	40	1882	0.08	25*	2.28×10^8^
**26695*nth***	53	1919	0.08	9	0.45
**26695*ung***	56	2390	0.60	7	0.18

Full data for all 18 mutants examined are contained in Tables S5–6. See Methods for interpretation of Bayes Factor values. Strongly significant results (Bayes factor ≥10) are marked with an asterisk.

1 Approximated using the Bayesian Information Criterion (cf. Methods).

**Figure 3:**
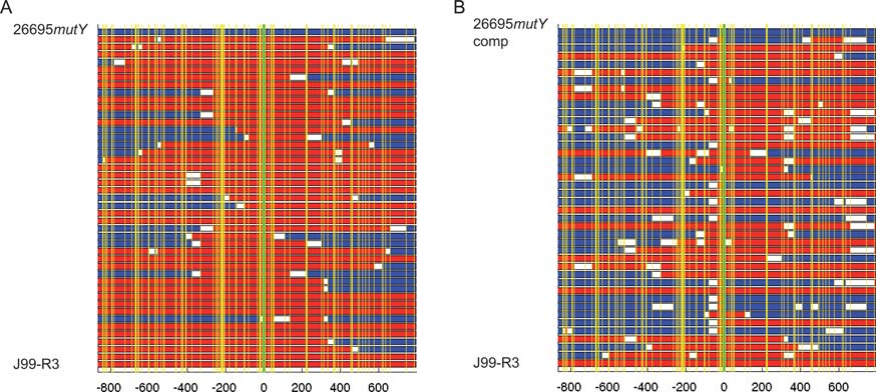
Effect of inactivation (strain 26695*mutY*, panel A) and overexpression (strain 26695*mutY*comp, panel B) of the DNA glycosylase gene *mutY* on import patterns. Color coding of sequences, see legend to Fig. 2.

To rule out that the increased import length was caused by polar effects of the insertion of the resistance cassette on genes downstream of *mutY*, we next performed a functional complementation experiment by introducing into the chromosome of the *mutY* mutant an intact copy of *mutY* that was under the control of the strong urease promoter [19]. The complemented strain, 26695*mutY*comp showed a ∼10 fold higher abundance of *mutY* transcript than the wild type strain 26695 (Fig. S1). As reported previously, the complementation restored the frequency of spontaneous mutations to Rif resistance to wild type level. The estimated mean import length of the complemented strain was 1882 bp, similar to the wild type length of 1681 bp, and strongly reduced in comparison to the import length of the *mutY* mutant (Table 3). Unexpectedly, the complemented strain also exhibited an increase in its frequency of ISR that was statistically significant, with 25 out of 40 recombinant clones containing ISR (Fig. 3).

Inactivation of two other DNA glycosylases in the *H. pylori* genome, *magIII* and *ung*, did not have a significant effect on import length or ISR.

## Discussion

The extent of allelic diversity within *H. pylori* has puzzled investigators since the late 1980s. Studies of population-wide nucleotide sequence variation, sequence comparisons of multiple strains isolated from a single biopsy, as well as comparisons of genetic relationships of sequential *H. pylori* isolates provided evidence that frequent recombination plays a central role in creating allelic diversity in *H. pylori* [7], [9], [20]. Studies of sequential isolates have also indicated that *H. pylori* imports short pieces of DNA with an estimated mean length of 417 bp into its chromosome after natural transformation [9]. However, systematic analyses of chromosomal import events after natural transformation have not been performed in *H. pylori*. In this study, we present extensive sequence data from a total of 1090 recombinant *H. pylori* clones resulting from transformation experiments with different combinations of recipients and donors, and investigated the roles of a large set of DNA recombination and repair genes in determining the length of imports after natural transformation.

### Length of import events

The estimated mean length of imports after transformation of *H. pylori* wild type strains ranged from 1294 to 3853 base pairs. While these values are still short in comparison with import length data reported from most other bacterial species, they exceeded those obtained by model-based analysis of sequences from sequential *H. pylori* isolates (417 bp, ref. [9]). These results indicate that import of short fragments after natural transformation is due to intrinsic properties of the *H. pylori* recombination/repair machinery. However, they also point at the existence of additional mechanisms contributing to the even shorter import events observed *in vivo*. For example, the moderately acidic environment of the natural ecological niche of *H. pylori*, a thin zone within the gastric mucus layer with a pH of ∼5.5–6 [21], may damage naked double stranded DNA (e.g. by depurination) before it is taken up by the recipient cell. This and other hypotheses are currently under investigation in our laboratory.

The length of imported fragments varied significantly between different combinations of donor and recipient. We first hypothesized that these differences might have been a consequence of varying levels of sequence similarity between the *rpoB* alleles (sequence identity ranged between 96.2% and 94.6% for the combinations of *rpoB* tested). However, our results argue strongly against this hypothesis, because in some cases, import lengths were significantly different when combinations of donor and recipient were reversed (e.g. N6/26695-R1 and 26695/N6-R1). Therefore, the mechanisms responsible for the differences of import length remain unknown, although our data suggest that import length is mainly determined by properties inherent to the recipient strains, such as their content of restriction modification systems. The distance of the import border to the central point mutation followed a geometric distribution, yielding no evidence that the location of the starting/end point of the import depended on specific sequence motifs.

Since *H. pylori* imports DNA through an unusual type IV secretion system (ComB, ref. [5]), we hypothesized that this machinery might be involved in generating short import events. However, this also seems unlikely, because the import length did not change when DNA was introduced into a ComB-deficient mutant by electroporation.

### Interspersed sequences of the recipient (ISR)

One striking feature of the imports we observed in *H. pylori* was the occurrence of short stretches of recipient sequence interspersed within the imported regions. The mean length of ISR was 82 bp. While these disruptions of the imported alleles were observed only in ∼10% of clones, ISR are likely to have an impact on allelic diversity, because they have the potential to significantly decrease linkage between individual polymorphic sites. *H. pylori* is characterized by a uniquely low linkage disequilibrium [7], and ISR are likely to contribute strongly to this allelic diversity, together with an overall high rate of recombination and a relatively short length of imported fragments.

The formation of complex mosaic alleles has previously been observed after transformation or transduction of homeologous DNA into *E. coli.* Abastado *et al*. obtained multiple different sequences with “patchwork structure” after transformation of *E. coli* with plasmids containing a heteroduplex [22], [23]. These mosaics were obtained in wild type as well as in *recA*-deficient recipients, suggesting that their formation resulted from repair processes rather than recombination. Since mosaicism was detected with oligonucleotide probes, detailed analysis of the imported sequences could not be performed at that time. McKane and Milkman used restriction fragment polymorphisms to characterize import events after transduction of ∼100 kb of DNA into *E. coli*, reporting that import frequently occurred in multiple fragments with a mean size of 8–14 kb. Similar to our data, fragment lengths were dependent upon the recipient strain used, and this was thought to be due to differences in the restriction modification systems [11]. To our knowledge, the present study is the first to use nucleotide sequencing of a large number of recombinant clones to analyze import patterns resulting from transformation. It is thus not possible to conclude with certainty whether ISR similar to those observed with *H. pylori* occur in other species, and at which frequency.

Formation of ISR might occur randomly at any position within the transformed fragment, or depend on the occurrence of specific sequence motifs, such as restriction sites. We tested the existence of such associations for 36 restriction sites within the *rpoB* sequence, and found no significant association between restriction sites in the *rpoB* fragment and the occurrence of ISR (data not shown). However, due to the relatively short length of the sequenced fragment, this analysis does not rule out the existence of such associations. Many restriction sites are present only once or twice in the sequenced fragment, making our dataset lowly informative about the association of ISR with particular sequence motifs.

We note that there was a significant increase in the number of clones with ISR when DNA was introduced by electroporation. Whether this is due to an increase of DNA repair activities after electroporation, damage to DNA generated by the pulse, the non-selective process by which the DNA is introduced into the cell by electroporation, or yet other mechanisms remains to be investigated.

To address the question whether ISR occur *in vivo*, we reanalyzed mosaic nucleotide sequences from sequential *H. pylori* isolates that were previously shown to have undergone recombination *in vivo* [9]. These paired sequences contain multiple events where blocks of sequence polymorphisms are interrupted by stretches of identical sequence (e.g. the *atpA* alleles of the pair NQ352). These patterns likely represent ISR, but it is impossible to prove this with certainty, because only recipient and recombinant sequences are known, whereas the donor sequence is not available, which would also be required to establish that the mosaic patterns are indeed ISR.

### A screen for genes controlling import length and ISR: the role of *mutY*

In order to identify genes that are involved in the control of import length and ISR formation, 18 candidate genes were selected. Mutants in these genes were constructed by allelic disruption and characterized with respect to import length and ISR, as well as mutation and recombinant frequencies (Tables S4, S5, S6, S7). This screen identified only one gene, *mutY*, whose inactivation affected import length and whose overexpression increased ISR frequency.

**Figure 4:**
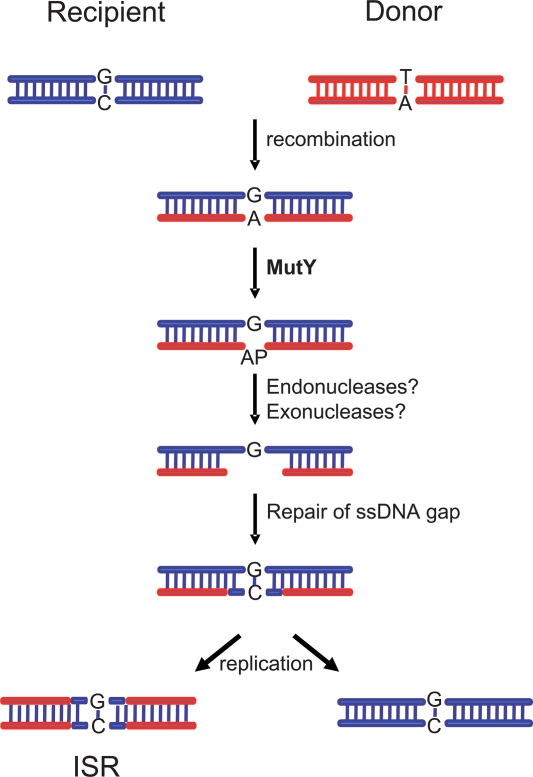
Hypothetical model of the involvement of MutY in the development of a short ISR in *H. pylori*. After the recombination of donor DNA (red) into the genome of a *H. pylori* recipient strain (blue) the resulting heteroduplex DNA molecule contains DNA mispairs at sites that differ between the two strains. Some of these mismatches (e.g. G:A or C:A basepairs) are recognized by the MutY glycosylase which subsequently hydrolyzes the N-glycosylic bond between the adenine (A) and the deoxyribose resulting in an apurinic site (AP) in one DNA strand. The AP site initiates further processes including endonuclease and/or exonuclease activities, leading to a single stranded DNA gap. The gap is closed by a polymerase and ligase and the mismatch is repaired. After replication, this gives rise to one daughter cell with recipient sequence and one carrying an ISR sequence.

The DNA glycosylase MutY is part of the base excision repair (BER) pathway and has a strong antimutator function in *H. pylori* [16], [18], [19], [24]. Unexpectedly, a *mutY* mutant exhibited a significantly increased import length compared with the wild type strain, while the frequency of ISR was not significantly affected. A functional complementation experiment was performed to rule out polar effects. Since the intact copy of *mutY* introduced in this strain is under control of a strong promoter [19], the complemented strain contains more *mutY* transcript than the wild type strain (Fig. S1). This complementation reduced the import length back to levels not distinguishable from wild type. Strikingly, it also caused a strong and highly significant increase in the number of ISR, suggesting that the MutY enzyme may be involved in initiating ISR formation after transformation. An involvement of MutY in recombination has been demonstrated before, albeit in a very different model system [25]. A hypothetical model of MutY-mediated formation of a short ISR is shown in Fig. 4. The inactivation of three other predicted BER glycosylase genes, *magIII*, *ung*, and *nth*, had no significant effect on the import length, and ISR were still observed in these mutants, indicating that neither of them is essential for ISR formation. We note that all single and double mutants studied were still capable of generating ISR. This may suggest that ISR formation can occur via multiple partially redundant repair pathways, and complete abrogation of ISR might require inactivation of multiple repair genes.

### Conclusion

The combination in *H. pylori* of competence for natural transformation, and high degree of sequence divergence between naturally occurring alleles of *rpoB* has enabled us to perform an in depth study of import events after transformation, and to identify the formation of mosaic sequences with interspersed recipient fragments as a mechanism likely contributing to the uniquely high allelic diversity in *H. pylori*. The experimental setup has also permitted to identify the glycosylase MutY as one gene involved in the control of import length and initiation of ISR formation. Our system will provide a powerful tool for further studies of recombination and possibly gene conversion in *H. pylori*, and should also be applicable to other transformable bacteria where many fundamental questions about the effect of recombination on the chromosome remain to be addressed.

## Materials and Methods

### Bacterial strains and culture conditions

Bacterial strains used in this study are listed in supporting information [SI] table S1 (references cited in supporting tables S1, S2, S3 are listed in Supplementary References S1). *Helicobacter pylori* wild type strains 26695 [26], J99 [27], and N6 [28] were cultured from frozen stocks on blood agar plates (Columbia agar base II, Oxoid, Wesel, Germany) containing 10% horse blood and antibiotics (vancomycin [10 mg/l], polymyxin B [2500 U/l], amphotericin B [4 mg/l], and trimethoprim [5 mg/l]). Plates were kept in an incubator with 5% O_2_, 10% CO_2_ and 85% N_2_ at 37°C for 24 h - 48 h. Mutant strains were cultivated on blood agar plates containing kanamycin (20 µg/ml), chloramphenicol (20 µg/ml), or both antibiotics as required. For selection of rifampicin (Rif) resistant *H. pylori* donor strains, 10^8^ wild type bacteria were plated on blood agar plates supplemented with rifampicin (10 µg/ml). Liquid cultures were performed in brain heart infusion (BHI, Oxoid) media with yeast extract (2.5 g/l), 10% heat inactivated horse serum and antibiotics (see above) in microaerobic atmosphere using anaerobic jars (Oxoid) and Anaerocult® C gas generating bags (Merck).

*E. coli* strains DH5α [29] and MC1061 [30] were used for the DNA cloning experiments and were grown in LB broth or on LB plates (Lennox L Broth, Invitrogen GmbH, Karlsruhe, Gemany) supplemented with ampicillin (200 µg/ml), chloramphenicol (20 µg/ml) and/or kanamycin (20 µg/ml) as required.

### DNA techniques

All standard cloning and DNA amplification, purification and manipulation procedures were done according to standard protocols [31]. Preparation of genomic DNA was performed using the QIAamp DNA Minikit (QIAGEN, Hilden, Germany). Large-scale purification of DNA was performed using QIAGEN Genomic-tip 100/G columns. Plasmid DNA from *E. coli* strains were isolated using QIAGEN tip 100 columns.

### Insertion mutagenesis in *H. pylori*

Construction of mutants by natural transformation mediated allelic exchange was performed as described previously [28], [32]. A list of the oligonucleotides used for mutagenesis, with introduced restriction sites is provided in Table S2. Briefly, the target genes were amplified by PCR and cloned into pUC18. The resulting plasmids (Table S3) were used for inverse PCR amplification with primers containing restriction sites. Inverse PCR reactions were designed in order to delete a part of the target gene and to introduce a unique restriction site. The PCR products were subsequently digested with the appropriate restriction enzyme, and ligated with an antibiotic resistance cassette (*aphA3*′-III or *cat*, refs. [33], [34] flanked by compatible restriction sites. The direction of transcription of the antibiotic resistance gene was the same as that of the target gene to avoid possible polar effects caused by the strong promoters of the antibiotic resistance cassettes.

Plasmids containing the interrupted gene were used as suicide plasmids in natural transformations of the *H. pylori* strains 26695, J99, or N6. The successful chromosomal replacement of the intact target gene with the disrupted gene construct via allelic exchange (double crossover) was checked by PCR using suitable primer combinations. For *mutY*, whose inactivation caused significant changes of the length of imported fragments, we performed a functional complementation experiment.

### *In vitro* transformation system of *H. pylori*

The transformation of *H. pylori* recipient strains with genomic DNA from Rif resistant donors was performed in 10 ml BHI including yeast extract (2.5 g/l), 10% horse serum and antibiotics (vancomycin [10 mg/l], polymyxin B [2500 U/l], amphotericin B [4 mg/l] and trimethoprim [5 mg/l]).

*H. pylori* wild type or mutant strains were grown for 24 h on non selective (for wild type strains) or selective (kanamycin and/or chloramphenicol as required for each knock out mutant) blood agar plates, as described above and harvested in 1.5 ml BHI broth. For strains J99, 26695 and N6, a suspension with an OD_600_ of 1 contains approx. 3×10^8^ bacteria [16]. The suspension was diluted to a start concentration of 2.1×10^7^ bacteria/ml in a final volume of 10 ml. For cultures containing knock-out mutant strains, kanamycin and/or chloramphenicol (each at 20 µg/ml) were added as required. The samples were then incubated for 20–24 h at 37°C in a rotary shaker (175 rpm) under microaerobic conditions using Anaerocult C bags (Merck). The bacterial concentration of the preparatory culture was measured and the suspension was used to start the experiment. Three 10 ml cultures per strain were adjusted to 1.5×10^7^ bacteria/ml and cultivated for 16 h as described above to achieve a concentration of approx. 3×10^8^ bacteria/ml. At this point, donor DNA (1 µg/ml) was added to two of the three cultures. All three cultures were then further incubated for 8 h. The OD_600_ was measured and for each culture incubated with donor DNA, inocula corresponding to 10^8^, 10^7^, and 10^6^ bacteria were plated on selective blood agar plates containing rifampicin (10 µg/ml) and cultivated for 5 days at 37°C. In parallel, a volume corresponding to 10^8^ bacteria from the recipient cultures cultivated without donor DNA were plated on selective Rif containing blood agar plates to determine the frequency of spontaneous Rif resistant mutants.

From each experiment, at least 16 clones were expanded in order to sequence a fragment (1663 bp) of the *rpoB* gene (see below). These experiments were reproduced at least 10 times for each *H. pylori* wild type strain and 3 times for each *H. pylori* mutant strain.

### Electroporation of *H. pylori*

For electroporation experiments, bacteria were grown to a density of 3×10^8^ cells per ml in 10 ml liquid cultures as described above, centrifuged and suspended in 10% glycerol (1 ml). 90 µl of the suspension were mixed with 10 µl of DNA in prechilled 0.2 cm electroporation cuvettes. Electroporation was performed at 2.5 kV, 25 µF and 400 Ω in a Gene Pulser (Biorad). After electroporation, the bacteria were added to 2 ml BHI media with 10% horse serum and yeast extract and incubated for 8 h. Plating and counting were done as described above.

### Partial sequence analysis of *rpoB* from *H. pylori* transformants

To determine the position of point mutations or import events in *rpoB*, a 2370 bp PCR fragment of *rpoB* was amplified with primers HPrpoB-1 and HPrpoB-6. This PCR product was used as the template for sequencing reactions with the primers HPrpoB-3, -4, -5, -6, -9w, and -10. Sequence reactions were done using the BigDye Terminator Cycle Sequencing Kit v1.1 (Applied Biosystems, Foster City, USA), and analyzed with the ABI 3130*xl* genetic analyzer (Applied Biosystems). The six sequences from each rifampicin resistant clone were assembled using the software Bionumerics V 4.5 (Applied Maths, Sint-Martens-Latem, Belgium), yielding a continuous, double-stranded 1663 bp fragment of *rpoB* that included the Rif resistance mediating point mutation of the donor strains.

### PCR-based prescreening for clones with DNA imports in the strains 26695*mutY* and 26695*ruvB*

Since it was difficult to find DNA imports in the strain 26695*mutY* due to the high background of spontaneous mutations and extraordinary low recombination levels in 26695*ruvB*, it was necessary to screen the Rif resistant clones after transformation by an import specific PCR using the primers HPrpoB-IscrX and HPrpoB-4. Primer HPrpoB-IscrX, specific for the Rif resistance mediating point mutation in strain J99-R3, was designed according to the method described by Furuta *et al*. [35]. PCR positive clones were subsequently used for sequencing as described above.

### Semi-quantitative reverse transcriptase PCR

Semi-quantitative RT-PCR was performed essentially as described previously [36]. Briefly, RNA was prepared from *H. pylori* strains grown in liquid culture to a density of 3×10^8^ bacteria/ml using the Qiagen RNeasy kit. Reverse transcription was performed using 2 µg of DNAse I treated RNA, a random hexamer primer mix and superscript III™ reverse transcriptase (Invitrogen, Karlsruhe, Gemany) at 42°C for 1 h. Semi-quantitative PCR of *mutY* was done with 2.5 µl cDNA in a total volume of 25 µl using the primers HPmutY1979 and HPmutY1980. To compare total amounts of RNA for different samples, a fragment of the 16S rRNA was amplified with the primer pair C05 and C97.

### Statistical methodology

Statistical analysis was performed by Bayesian model comparison, where two competing models are compared using the Bayes Factor (BF) which is equal to the ratio of probabilities of observing the data under each model (see refs. [37], [38] for reviews). This has the advantage to directly weight the evidence for two competing hypothesis, taking into account their relative complexity, so that over-complicated models are naturally penalized. Interpretation of the Bayes Factor was done following the guidelines of Jeffreys [39]: Negative (<1); Barely worth mentioning (≥1-<3); Substantial (≥3–10); Strong (≥10–30); Very strong (≥30-<100); Decisive (≥100).

When the Bayes Factor could not be explicitly calculated, a conservative estimate was computed using the Bayes Information Criterion (BIC; refs. [37], [40]

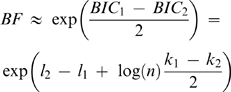
(1)where *l*_1_ and *l*_2_ are the maximized value of the log-likelihood under the two models, *k*_1_ and *k*_2_ the number of free parameters in the two models, and *n* the number of observations. Comparisons of frequency data between any two recipient/donor combinations were done using the BIC for two models: one where the data from the two combinations comes from the same Normal distribution and one where it comes from two distinct Normal distributions.

Import length is often modelled using a geometric distribution [9], [13], [41]. Using the BIC, we showed that this is a better model than a negative binomial model, despite its additional free parameter. We do not observe exactly where recombination starts and ends for an observation *i*, but instead an interval [*m_i_*; *M_i_*] where it might happen (with *M_i_* = ∞ if the beginning or end is out of the sequenced region), so that the likelihood of *N* observations under the geometric model with mean length δ is equal to:
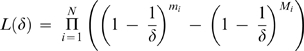
(2)Lengths of imports on either side of the resistance points for each recipient/donor combination were compared using the BIC with one model where left and right come from the same geometric distribution and one model where they do not. We found no evidence for a difference in length on left and right. The effect of gene knock-outs on the lengths of import was evaluated in the same way (comparing results between two combinations rather than between left and right of a single combination).

Let *p* denote the probability of occurrence of ISR in a sequenced clone. The number *m* of clones containing ISR amongst *n* sequenced clones is thus distributed as Binomial(*n*,*p*). Assuming a Jeffrey's prior on *p* (i.e. Beta(½,½)), we compared the value of *p* between two recipient/donor combinations (*m*_1_,*n*_1_ and *m*_2_,*n*_2_) using the Bayes Factor:
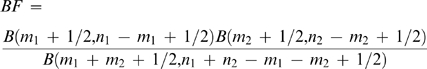
(3)where B(.,.) denotes the Euler Beta function.

## Supporting Information

Table S1 Bacterial strains used in this study
(0.05 MB PDF)

Table S2 Oligonucleotide primers and PCR products used in this study
(0.06 MB PDF)

Table S3 Plasmids used in this study
(0.02 MB PDF)

Table S4 Mutation and recombination frequencies leading to Rif resistance in *H. pylori* wild type strains and mutants
(0.03 MB PDF)

Table S5 Maximum likelihood estimation (MLE) of the mean length of DNA imports left and right from the Rif resistance mediating mutation.
(0.02 MB PDF)

Table S6 Clones with ISR observed in the analyzed *H. pylori* wild type and mutant strains.
(0.02 MB PDF)

Table S7 Maximum likelihood estimation (MLE) of the mean length of ISR in the analyzed *H. pylori* wild type and mutant strains
(0.02 MB PDF)

Supplementary References S1 References for supplementary material
(0.04 MB DOC)

Figure S1 Semi-quantitative RT-PCR analyses of the abundance of *mutY* mRNA in the *H. pylori* strains 26695 and 26695mutYcomp.
(0.12 MB PDF)
