# Supplementary material for: Mosaic DNA Imports with Interspersions of Recipient Sequence after Natural Transformation of Helicobacter pylori
Source: PLoS One. 2008 Nov 24;3(11):e3797. doi: 10.1371/journal.pone.0003797 (PMC2582958; doi:10.1371/journal.pone.0003797)
Supplement: Table S1 — Bacterial strains used in this study (0.05 MB PDF) [file pone.0003797.s001.pdf]

S. Kulick et al.

Supporting information

Table S1: Bacterial strains

Strain	Genotype	Source
Escherichia coli		
DH5α	F^-, φ80d*lacZ*ΔM15, Δ(*lacZYA-argF*)U169, *deoR*, *recA*1, *endA*1, *hsdR*17(rk$^-$, mk$^+$), *phoA*, *supE*44, λ$^-$, *thi*-1, *gyrA*96, *relA*1	(S1)
MC1065	*E. coli* K-12 *leu*B6 Δ(*lac*JPOZY)X74 *trpC*9830 *strA*	(S2)
Helicobacter pylori		
26695	*H. pylori* wild type strain	(S3)
J99	*H. pylori* wild type strain	(S4)
N6	*H. pylori* wild type strain	(S5)
26695-R1	Derivative of 26695; T to C mutation at position 1757 in *rpoB*; Rif^r	This study
J99-R3	Derivative of J99; A to T mutation at position 1618 in *rpoB* ; Rif^r	This study
N6-R1	Derivative of N6; C to T mutation at position 1589 in *rpoB* ; Rif^r	This study
26695*comB10*	HP0042 (DNA transformation competence protein) from wt strain 26695 inactivated with *aphA3*'-III	This study
26695*magIII*	HP0602 (3-Methyladenine glycosylase) from wt strain 26695 inactivated with *aphA3*'-III	This study
26695*mfd*	HP1541 (Transcription repair coupling factor) from wt strain 26695 inactivated with *cat*	This study
26695*mutS*	HP0621 (Predicted homologous and homeologous recombination protein) from wt strain 26695 inactivated with *aphA3*'-III	This study
26695*mutY*	HP0142 (A/G-specific adenine glycosylase) from wt strain 26695 inactivated with *aphA3*'-III	(S6)
26695*mutY*comp	26695*mutY* strain complemented with pADC-HpMutY plasmid	This study
26695*nth*	HP0585 (Endonuclease III) from wt strain 26695 inactivated with *cat*	This study
26695*nucT*	HP0323 (Endonuclease) from wt strain inactivated with *aphA3*'-III	This study
26695*recA*	HP0153 (Recombinase) from wt strain 26695 inactivated with *aphA3*'-III	This study
26695*recB*	HP1553 (*recB* homologue) from wt strain 26695 inactivated with *cat*	This study
26695*recG*	HP1523 (Helicase) from wt strain 26695 inactivated with *cat*	This study

26695*recJ*	HP0348 (single-stranded-DNA-specific exonuclease) from wt strain 26695 inactivated with *aphA3*'-III	This study
26695*recJ xseA*	HP0348 and JHP0243 (exonucleases) inactivated with *aphA3*'-III and *cat*, respectively	This study
26695*recN*	HP1393 (Predicted homologous recombination protein) from wt strain 26695 inactivated with *cat*	This study
26695*recR*	HP0925 (Predicted recombinational DNA repair protein) from wt strain 26695 inactivated with *cat*	This study
26695*ruvA*	HP0883 (Juction targeting protein) from wt strain 26695 inactivated with *aphA3*'-III	This study
26695*ruvB*	HP1059 (Helicase) from wt strain 26695 inactivated with *aphA3*'-III	This study
26695*ruvC*	HP0877 (Resolvase) from wt strain 26695 inactivated with *aphA3'*-III	This study
26695*ung*	HP1347 (Uracil-DNA glycosylase) from wt strain 26695 inactivated with *cat*	This study
26695*xseA*	JHP0243 (exonuclease VII, large subunit) from wt strain J99 inactivated with *cat*	This study
26695*xth*	HP1526 (Exonuclease III) from wt strain 26695 inactivated with *aphA3*'-III	This study
