# Supplementary material for: Mosaic DNA Imports with Interspersions of Recipient Sequence after Natural Transformation of Helicobacter pylori
Source: PLoS One. 2008 Nov 24;3(11):e3797. doi: 10.1371/journal.pone.0003797 (PMC2582958; doi:10.1371/journal.pone.0003797)
Supplement: Table S2 — Oligonucleotide primers and PCR products used in this study (0.06 MB PDF) [file pone.0003797.s002.pdf]

**S. Kulick et al.**

**Supporting information**

Table S2: Oligonucleotide primers and PCR products used in this study

| **Primer** | **Target gene** | **5' → 3' sequence** | **RS** | **Appli-cation** | **Source** |
|---|---|---|---|---|---|
| C05 | 16S rDNA | ACTTCACCCCAGTCGCTG | | RT-PCR | (S7) |
| C97 | | GCTATGACGGGTATCC | | | |
| HPcomB10-1s | HP0042 (*comB10*) | gcgctgcagGTGAATAAGTGGATTAAAGG | PstI | Cloning | This study |
| HPcomB10-2s | | gcgctgcagCGATGACTTCATTCTCTCTG | PstI | | |
| HPcomB10-3s | | ataagatctCTGTAATGGTGCGTAAAAGC | BglII | Inverse PCR | |
| HPcomB10-4s | | ttaagatctGATTACGCCCATTTCTAGCC | BglII | | |
| HPmagIII-1s | HP0602 (*magIII*) | ataggatccGCTATGCGATGAGTCAAGCC | BamHI | Cloning | This study |
| HPmagIII-2s | | aatggatccAATCGCCCATTCCTGCATCC | BamHI | | |
| HPmagIII-3s | | ataagatctCAAATCAATCAGTCGTTTGGC | BglII | Inverse PCR | |
| HPmagIII-4s | | ttaagatctAAGAAGTAACCAGACAGTGG | BglII | | |
| HPmfd-1PstI | HP1541 (*mfd*) | gcgctgcagTCCGAGCTCGCTAAAGAAGTC | PstI | Cloning | This study |
| HPmfd-2PstI | | gcgctgcagGGTTGGCTAGAATTTTAAGC | PstI | | |
| HPmfd-3BglII | | ataagatctTCTTTAGCTGGCACGCTATC | BglII | Inverse PCR | |
| HPmfd-4BglII | | ataagatctGTTTGAATACACCAGCGATC | BglII | | |
| HPmutS-1s | HP0621 (*mutS*) | ataggatccACTCACCAAATTAGGGACGC | BamHI | Cloning | This study |
| HPmutS-2s | | ataggatccTCCAGTAGAGATATTGTTCGCGC | BamHI | | |
| HPmutS-3s | | ataagatctGCTTTAGCGAAATTAAGCCG | BglII | Inverse PCR | |
| HPmutS-4s | | ataagatctAGAATTTGTCATGCCAAGC | BglII | | |
| HPmutY-1 | HP0142 (*mutY*) | gcgctgcagTTTGGGCAAGATGATAAGCC | PstI | Cloning | (S6) |
| HPmutY-2 | | gcgctgcagGTGGTTGTAGTTGGAAACTTTAC | PstI | | |
| HPmutY-3 | | ataagatctAAGCTTTAATGATAACCAAGCC | BglII | Inverse PCR | |
| HPmutY-4 | | aatagatctAAGATTGAGAAAGTCATTCGC | BglII | | |
| HPmutY1979 | | GTGGTTGTAGYTGGAAACTTTACAC | | RT-PCR | http://pubmlst.org/helicobacter/ |
| HPmutY1980 | | CAACGCCCAAGTAACGCTCTTC | | | |
| HPnth-1PstI | HP0585 (*nth*) | gcgctgcagCGTGAATGTCAAAGGCACAC | PstI | Cloning | This study |
| HPnth-2PstI | | gcgctgcagAAAGTATCCACGCTCTCTCC | PstI | | |
| HPnth-3BglII | | ttaagatctGAGGGCTAAATCGTTCACGC | BglII | Inverse PCR | |
| HPnth-4BglII | | ttaagatctATGTGGTGCTTTCAGTGTGC | BglII | | |
| HPnucT-1s | HP0323 (*nucT*) | ataggatccATCATCGGCTATCAAAAGCC | BamHI | Cloning | This study |
| HPnucT-2s | | aatggatccGGAATAGCTGGTATATTGGC | BamHI | | |

| | | | | | |
|---|---|---|---|---|---|
| HPnucT-3s | | ataagatctATAGCCAATAGTGGATTGC | BglII | Inverse PCR | |
| HPnucT-4s | | ttaagatctACCCTAACACGAAAGTGTGC | BglII | | |
| HP1553-1PstI | HP1553 (*recB*) | gcgctgcagGAATTTGACGCTGTCTTTGC | PstI | Cloning | This study |
| HP1553-2PstI | | gcgctgcagTCTTAAGCGTCAAACTGAGC | PstI | | |
| HP1553-3BclI | | atagcgtgatcaAATGATTAAAGGCGCACTGC | BclI | Inverse PCR | |
| HP1553-4BclI | | atagcgtgatcaTTTATGCGCCACTAATGACG | BclI | | |
| HPrecA-1s | HP0153 (*recA*) | ataggatccGGCAATAGATGAAGACAAAC | BamHI | Cloning | This study |
| HPrecA-2s | | aatggatccACTCCATTTCTTCTAAAGGC | BamHI | | |
| HPrecA-3s | | ataagatctCGTCATGCCAATCTTCATTC | BglII | Inverse PCR | |
| HPrecA-4s | | ttaagatctGGAGTCCAGAGACTACAAC | BglII | | |
| HP1523-1PstI | HP1523 (*recG*) | gcgctgcagAGGCTCGTCTTTGCATGC | PstI | Cloning | This study |
| HP1523-2PstI | | gcgctgcagTTAAATTACCCAATTCTGCC | PstI | | |
| HP1523-3BglII | | ataagatctTTAAGGCTTCGTTATAAAGC | BglII | Inverse PCR | |
| HP1523-4BglII | | ataagatctAGCGTTGTTGTTTGATAAGC | BglII | | |
| HPrecJ-1s | HP0348 (*recJ*) | gcgctgcagAAGAGCGCATGGCTTCTATC | PstI | Cloning | This study |
| HPrecJ-2s | | gcgctgcagACACTCATCTAATTCCACGC | PstI | | |
| HPrecJ-3s | | ataagatctCCCTGCGGACTTGATTAAGG | BglII | Inverse PCR | |
| HPrecJ-4s | | ttaagatctATGCAAGATGCGAAAATGGC | BglII | | |
| HP1393-1PstI | HP1393 (*recN*) | gcgctgcagATGAAGACGCTCTTGTGGTC | PstI | Cloning | This study |
| HP1393-2PstI | | gcgctgcagATATAAGGTGCGCTCAAATCTAC | PstI | | |
| HP1393-3BglII | | ataagatctCGCTCTCTAAAGCATGCGTG | BglII | Inverse PCR | |
| HP1393-4BglII | | ataagatctTTGGCATGCTAAGTGGGATC | BglII | | |
| HP0925-1PstI | HP0925 (*recR*) | gcgctgcagTTTGTTGGCCCTCCATTTTC | PstI | Cloning | This study |
| HP0925-2PstI | | gcgctgcagAATTAGGCATTCCAAACTGC | PstI | | |
| HP0925-3BglII | | ataagatctTTGTCGGCTTTCATCAGAGC | BglII | Inverse PCR | |
| HP0925-4BglII | | ataagatctCCCACTTTAGCTAATGATTC | BglII | | |
| HPruvA-1PstI | HP0883 (*ruvA*) | gcgctgcagCTCAGCACCAGTCCTTAAAC | PstI | Cloning | This study |
| HPruvA-2PstI | | gcgctgcagTGTTTGGTGCTCCGTAATGC | PstI | | |
| HPruvA-3ClaI | | ccatcgatggAATGGCTAAAGCGATACGCC | ClaI | Inverse PCR | |
| HPruvA-4ClaI | | ccatcgatggAAGATGAAAACAGACCCGC | ClaI | | |
| HPruvB-1PstI | HP1059 (*ruvB*) | gcgctgcagTGTGGAGCGATATGTTAGGC | PstI | Cloning | This study |
| HPruvB-2PstI | | gcgctgcagAACTTCACCACATCCACGAC | PstI | | |
| HPruvB-3BglII | | ataagatctCTCTTAAGGGGTTAGAGAGC | BglII | Inverse PCR | |
| HPruvB-4BglII | | ataagatctGCTAAAAGGAGTAGAGGCAC | BglII | | |
| HPruvC-1PstI | HP0877 (*ruvC*) | gcgctgcagCCATTTAGTCGCTTCACTGC | PstI | Cloning | This study |
| HPruvC-2PstI | | gcgctgcagTCAATCTTGCATGGCTTAGC | PstI | | |

| | | | | | |
|---|---|---|---|---|---|
| HPruvC-3ClaI | | ccatcgatggTCTGTTCTTGCAAGCGTGTC | ClaI | Inverse PCR | |
| HPruvC-4ClaI | | ccatcgatggTAGCGAATACACGCCCTTAC | ClaI | | |
| HPung-1PstI | HP1347 (*ung*) | gcgctgcagGGCATGCCTTAACGGATACC | PstI | Cloning | This study |
| HPung-2PstI | | gcgctgcagAAATGCCCATGCACGCTGTC | PstI | | |
| HPung-3BglII | | ttaagatctGCCACCGGCAATTCTTGATC | BglII | Inverse PCR | |
| HPung-4BglII | | ttaagatctCTTGTTGTGGGGATTTGAGC | BglII | | |
| HPxseA-1s | HP0259 | gcgctgcagATGCATGTATTGAGCGTGAGC | PstI | Cloning | This study |
| HPxseA-2s | JHP0243 (*xseA*) | gcgctgcagCTACACCCTATCCACGCTC | PstI | | |
| HPxseA-3s | | ataagatctTGATGCAAACCAATTCGCAC | BglII | Inverse PCR | |
| HPxseA-4s | | ttaagatctTTCAAAGCGTGGTGGAGAGC | BglII | | |
| HP1526-1s | HP1526 (*xth*) | gcgctgcagCAACGAAGTTTAAGATGGTGG | PstI | Cloning | This study |
| HP1526-2s | | gcgctgcagGCAGCATTCAAGGGTTTTCG | PstI | | |
| HP1526-3s | | ataagatctAAAACGACTCAAATTCGCAAG | BglII | Inverse PCR | |
| HP1526-4s | | ttaagatctCTATCCAGGCTTAGTTATCG | BglII | | |
| pcat-1 | *cat* cassette | AACAGCTATGACCATGATTACG | | Cloning | (S7) |
| pCAT-12NdeI | | agacatatgGATATCGCATGCCTGCAGAG | NdeI | | |
| pcat-2 | | agaggatccGATATCGCATGCCTGCAGAG | BamHI | | |
| HPrpoB-1 | *rpoB* (HP1198) | CCCAACAGATTTAGAAGT | | *rpoB* sequen-cing | This study |
| HPrpoB-3 | | ATGTGCCTGATTACATCACGAC | | | |
| HPrpoB-4 | | TTGGCGCTGCATGTTAGTCC | | | |
| HPrpoB-5 | | GGTAGCCGCATCGCTCATTC | | | |
| HPrpoB-6 | | TTCCCTAACGCTAACTCGC | | | |
| HPrpoB-9w | | AGACGCYAATCARAGAATGG | | | |
| HPrpoB-10 | | CATCAATCTTGCCCTGATTG | | | |
| HPrpoB-IscrX | *rpoB* | CCTTTGAGTGAAGTTCCGTA | | Import scree-ning with rpoB-4 26695 (J99R3) combi-nation | This study |
