# Supplementary material for: Mosaic DNA Imports with Interspersions of Recipient Sequence after Natural Transformation of Helicobacter pylori
Source: PLoS One. 2008 Nov 24;3(11):e3797. doi: 10.1371/journal.pone.0003797 (PMC2582958; doi:10.1371/journal.pone.0003797)
Supplement: Table S3 — Plasmids used in this study (0.02 MB PDF) [file pone.0003797.s003.pdf]

**S. Kulick et al.**

**Supporting information**

Table S3: Plasmids used in this study

| **Plasmids** | **Genotype** | **Source** |
|---|---|---|
| pBHpC8 | Source of the *cat* cassette | (S7) |
| pILL 600 | Source of the *aphA3´-III* cassette | (S8) |
| pUC18 | $Amp^r$, Col×101, MCS within *lacZ*: blue/white selection | (S9) |
| pSUS1707 | $Amp^r$, pUC18 derivative containing *mutS2* | This study |
| pSUS1711 | $Amp^r$, $Km^r$, pSUS1707 derivative with a *mutS2::aphA3´-III* disruption | This study |
| pSUS2500 | $Amp^r$, pUC18 derivative containing *mutY* | (S6) |
| pSUS2501 | $Amp^r$, pUC18 derivative containing *comB10* | This study |
| pSUS2502 | $Amp^r$, pUC18 derivative containing *recJ* | This study |
| pSUS2503 | $Amp^r$, pUC18 derivative containing *recA* | This study |
| pSUS2508 | $Amp^r$, $Km^r$, pSUS2500 derivative with a *mutY::aphA3-III* disruption | (S6) |
| pSUS2509 | $Amp^r$, $Km^r$, pSUS2501 derivative with a *comB10::aphA3´-III* disruption | This study |
| pSUS2510 | $Amp^r$, $Km^r$, pSUS2502 derivative with a *recJ::aphA3´-III* disruption | This study |
| pSUS2511 | $Amp^r$, $Km^r$, pSUS2503 derivative with a *recA::aphA3´-III* disruption | This study |
| pSUS2518 | $Amp^r$, pUC18 derivative containing *xth* | This study |
| pSUS2520 | $Amp^r$, $Km^r$, pSUS2518 derivative with a *xth::aphA3´-III* disruption | This study |
| pSUS2524 | $Amp^r$, pUC18 derivative containing *xseA* | This study |
| pSUS2525 | $Amp^r$, $Cm^r$, pSUS2524 derivative with a *xseA::cat* disruption | This study |
| pSUS2526 | $Amp^r$, pUC18 derivative containing *recR* | This study |
| pSUS2527 | $Amp^r$, pUC18 derivative containing *recN* | This study |
| pSUS2528 | $Amp^r$, pUC18 derivative containing *recG* | This study |
| pSUS2529 | $Amp^r$, pUC18 derivative containing *recB* homologue | This study |
| pSUS2530 | $Amp^r$, $Cm^r$, pSUS2526 derivative with a *recR::cat* disruption | This study |
| pSUS2532 | $Amp^r$, $Cm^r$, pSUS2527 derivative with a *recN::cat* disruption | This study |
| pSUS2534 | $Amp^r$, $Cm^r$, pSUS2528 derivative with a *recG::cat* disruption | This study |

| | | |
|---|---|---|
| pSUS2535 | Amp$^r$, Cm$^r$, pSUS2529 derivative with a *recB::cat* disruption | This study |
| pSUS2537 | Amp$^r$, pUC18 derivative containing *nth* | This study |
| pSUS2538 | Amp$^r$, pUC18 derivative containing *ung* | This study |
| pSUS2540 | Amp$^r$, Cm$^r$, pSUS2537 derivative with a *nth::cat* disruption | This study |
| pSUS2541 | Amp$^r$, Cm$^r$, pSUS2538 derivative with a *ung::cat* disruption | This study |
| pSUS2600 | Amp$^r$, pUC18 derivative containing *magIII* | This study |
| pSUS2602 | Amp$^r$, Km$^r$, pSUS2600 derivative with a *magIII::aphA3´-III* disruption | This study |
| pSUS2604 | Amp$^r$, pUC18 derivative containing *nucT* | This study |
| pSUS2606 | Amp$^r$, Km$^r$, pSUS2604 derivative with a *nucT::aphA3´-III* disruption | This study |
| pSUS2613 | Amp$^r$, pUC18 derivative containing *mfd* | This study |
| pSUS2615 | Amp$^r$, Cm$^r$, pSUS2613 derivative with a *mfd::cat* disruption | This study |
| pSUS2616 | Amp$^r$, pUC18 derivative containing *ruvA* | This study |
| pSUS2618 | Amp$^r$, Km$^r$, pSUS2616 derivative with a *ruvA::aphA3´-III* disruption | This study |
| pSUS2619 | Amp$^r$, pUC18 derivative containing *ruvB* | This study |
| pSUS2621 | Amp$^r$, Km$^r$, pSUS2619 derivative with a *ruvB::aphA3´-III* disruption | This study |
| pSUS2622 | Amp$^r$, pUC18 derivative containing *ruvC* | This study |
| pSUS2624 | Amp$^r$, Km$^r$, pSUS2622 derivative with a *ruvC::aphA3´-III* disruption | This study |
| pADC-HpMutY | Amp$^r$, Cm$^r$, *ureAB* fragment in pUC18 with *H. pylori mutY* and *cat* cassette | (S10) |
