# Supplementary material for: Mosaic DNA Imports with Interspersions of Recipient Sequence after Natural Transformation of Helicobacter pylori
Source: PLoS One. 2008 Nov 24;3(11):e3797. doi: 10.1371/journal.pone.0003797 (PMC2582958; doi:10.1371/journal.pone.0003797)
Supplement: Table S4 — Mutation and recombination frequencies leading to Rif resistance in H. pylori wild type strains and mutants (0.03 MB PDF) [file pone.0003797.s004.pdf]

S. Kulick et al.

Supporting information

Table S4: Mutation and recombination frequencies leading to Rif resistance in *H. pylori* wild type strains and mutants

		Frequencies without donor DNA			Frequencies with donor DNA		
**Recipient**	**Donor**	**Mean[1]**	**Std**	**Bayes Factor[2]**	**Mean[1]**	**Std**	**Bayes Factor[2]**
**26695**	**J99-R3**	1.14×10^{-06}	4.95×10^{-07}		4.17×10^{-05}	5.37×10^{-05}	
	**N6-R1**				2.78×10^{-05}	7.16×10^{-06}	12.27
**J99**	**26695-R1**	1.56×10^{-06}	1.86×10^{-06}	$8.23\times10^{+05}$	2.84×10^{-04}	3.28×10^{-04}	$3.24\times10^{+12}$
**N6**	**26695-R1**	4.56×10^{-07}	3.54×10^{-07}	$3.67\times10^{+03}$	1.01×10^{-05}	1.27×10^{-05}	2.30
	**J99-R3**				9.07×10^{-05}	1.61×10^{-04}	$1.16\times10^{+05}$
**26695 *comB10***	**J99-R3**	1.23×10^{-06}	1.15×10^{-06}	0.61	1.18×10^{-06}	1.10×10^{-06}	$1.97\times10^{+06}$
	**J99-R3EP**	9.95×10^{-07}	1.95×10^{-07}	0.08	1.40×10^{-05}	1.52×10^{-05}	1.01
**26695*magIII***	**J99-R3**	1.24×10^{-06}	3.37×10^{-07}	0.04	2.10×10^{-05}	1.97×10^{-05}	2.95
**26695*mfd***	**J99-R3**	1.05×10^{-06}	4.43×10^{-07}	0.03	6.18×10^{-05}	1.04×10^{-04}	1.06
**26695*mutS***	**J99-R3**	9.85×10^{-07}	7.95×10^{-07}	0.05	2.55×10^{-05}	3.08×10^{-05}	0.07
**26695*mutY***	**J99-R3**	2.93×10^{-05}	2.12×10^{-05}	$2.47\times10^{+47}$	4.65×10^{-05}	2.43×10^{-05}	0.24
**26695*mutY* comp**	**J99-R3**	4.37×10^{-07}	2.24×10^{-07}	1.31	1.83×10^{-06}	6.71×10^{-07}	$2.24\times10^{+07}$
**26695*nth***	**J99-R3**	9.73×10^{-06}	1.23×10^{-06}	$1.63\times10^{+23}$	2.21×10^{-04}	1.03×10^{-04}	$4.06\times10^{+05}$
**26695*nucT***	**J99-R3**	2.43×10^{-06}	1.81×10^{-06}	$1.34\times10^{+05}$	4.86×10^{-05}	6.43×10^{-05}	0.03
**26695*recA***	**J99-R3**	7.00×10^{-08}	7.00×10^{-08}	31.37	8.73×10^{-08}	7.34×410^{-08}	$2.99\times10^{+09}$
**26695*recB***	**J99-R3**	1.40×10^{-07}	9.90×10^{-08}	144.25	1.25×10^{-06}	1.07×10^{-06}	$8.02\times10^{+07}$
**26695*recG***	**J99-R3**	6.33×10^{-07}	$3.09\times10\text{-}^{07}$	0.18	1.62×10^{-04}	1.21×10^{-04}	$1.91\times10^{+03}$
**26695*recJ***	**J99-R3**	7.90×10^{-07}	6.04×10^{-07}	0.07	1.84×10^{-05}	1.52×10^{-05}	4.34
**26695*recJ* xseA**	**J99-R3**	1.48×10^{-06}	5.81×10^{-07}	0.05	1.33×10^{-04}	9.69×10^{-05}	28.80
**26695*recN***	**J99-R3**	5.44×10^{-07}	4.82×10^{-07}	1.14	3.95×10^{-06}	7.45×10^{-06}	$1.58\times10^{+08}$
**26695*recR***	**J99-R3**	8.60×10^{-07}	6.56×10^{-07}	0.05	3.46×10^{-04}	1.35×10^{-04}	$1.52\times10^{+11}$
**26695*ruvA***	**J99-R3**	7.50×10^{-08}	8.38×10^{-08}	$7.57\times10^{+03}$	1.72×10^{-06}	1.51×10^{-06}	$2.41\times10^{+08}$
**26695*ruvB***	**J99-R3**	5.05×10^{-07}	1.23×10^{-07}	18.81	1.50×10^{-06}	1.52×10^{-06}	$5.77\times10^{+09}$
**26695*ruvC***	**J99-R3**	1.10×10^{-07}	8.69×10^{-08}	$4.37\times10^{+03}$	5.39×10^{-06}	4.65×10^{-06}	$5.36\times10^{+05}$

**26695*ung***	**J99-R3**	2.43×10^{-06}	1.25×10^{-07}	$1.33\times10^{+03}$	2.08×10^{-05}	2.09×10^{-05}	0.75
**26695*xseA***	**J99-R3**	1.28×10^{-06}	4.65×10^{-07}	0.03	9.69×10^{-04}	6.60×10^{-04}	$1.01\times10^{+26}$
**26695*xth***	**J99-R3**	1.06×10^{-06}	2.24×10^{-07}	0.09	5.76×10^{-05}	8.87×10^{-05}	0.12

[1] Cells marked in green represent significant increases of the mutation or recombination frequencies in comparison to the recipient-donor combination 26695/J99-R3 (first row). Cells marked in red show significant decreases.

[2] Approximated using the Bayesian Information Criterion (cf. Methods).
