# Supplementary material for: Mosaic DNA Imports with Interspersions of Recipient Sequence after Natural Transformation of Helicobacter pylori
Source: PLoS One. 2008 Nov 24;3(11):e3797. doi: 10.1371/journal.pone.0003797 (PMC2582958; doi:10.1371/journal.pone.0003797)
Supplement: Table S5 — Maximum likelihood estimation (MLE) of the mean length of DNA imports left and right from the Rif resistance mediating mutation. (0.02 MB PDF) [file pone.0003797.s005.pdf]

S. Kulick et al.

Supporting information

Table S5: Maximum likelihood estimation (MLE) of the mean length of DNA imports left and right from the Rif resistance mediating mutation. Cells color-coded (See Table S4)

Recipient	Donor	Num clones[1]	MLE (bp)	Bayes Factor[2]
**26695**	**J99-R3**	**95**	**1681**	
	**N6-R1**	26	2434	0.29
**J99**	**26695-R1**	32	1294	0.19
**N6**	**26695-R1**	25	3819	28.21
	**J99-R3**	80	3853	$4.43 \times 10^{+04}$
**26695*comB10***	**J99-R3**	0	-	-
**26695*comB10* EP**	**J99-R3**	25	2228	0.15
**26695*magIII***	**J99-R3**	51	1729	0.06
**26695*mfd***	**J99-R3**	29	1939	0.08
**26695*mutS***	**J99-R3**	53	1639	0.06
**26695*mutY***	**J99-R3**	43	3268	38.88
**26695*mutY* comp**	**J99-R3**	40	1882	0.08
**26695*nth***	**J99-R3**	53	1919	0.08
**26695*nucT***	**J99-R3**	29	2926	2.19
**26695*recA***	**J99-R3**	0	-	-
**26695*recB***	**J99-R3**	51	2621	2.00
**26695*recG***	**J99-R3**	63	1912	0.08
**26695*recJ***	**J99-R3**	61	1989	0.11
**26695*recJxseA***	**J99-R3**	55	2348	0.50
**26695*recN***	**J99-R3**	59	2255	0.33
**26695*recR***	**J99-R3**	69	2026	0.12
**26695*ruvA***	**J99-R3**	41	2564	0.92
**26695*ruvB***	**J99-R3**	1	-	-
**26695*ruvC***	**J99-R3**	41	1691	0.06
**26695*ung***	**J99-R3**	56	2390	0.60
**26695*xseA***	**J99-R3**	59	1938	0.09
**26695*xth***	**J99-R3**	33	1696	0.06

[1] Num clones = number of clones with DNA imports in *rpoB.*

[2] Approximated using the Bayesian Information Criterion (cf. Methods).
