# Supplementary material for: Mosaic DNA Imports with Interspersions of Recipient Sequence after Natural Transformation of Helicobacter pylori
Source: PLoS One. 2008 Nov 24;3(11):e3797. doi: 10.1371/journal.pone.0003797 (PMC2582958; doi:10.1371/journal.pone.0003797)
Supplement: Table S6 — Clones with ISR observed in the analyzed H. pylori wild type and mutant strains. (0.02 MB PDF) [file pone.0003797.s006.pdf]

S. Kulick et al.

Supporting information

Table S6: Clones with ISR observed in the analyzed *H. pylori* wild type and mutant strains. Cell color-coded (See Table S4)

Recipient strain	Donor DNA	Num clones[1]	Clones with ISR	Bayes Factor[2]
**26695**	**J99-R3**	**95**	**8**	
	**N6-R1**	26	3	0.19
**J99**	**26695-R1**	32	4	0.20
**N6**	**26695-R1**	25	5	0.66
	**J99-R3**	80	8	0.12
**26695*comB10***	**J99-R3**	0	0	1.00
**26695*comB10* EP**	**J99-R3**	25	8	11.45
**26695*magIII***	**J99-R3**	51	9	0.53
**26695*mfd***	**J99-R3**	29	3	0.17
**26695*mutS***	**J99-R3**	53	10	0.75
**26695*mutY***	**J99-R3**	43	9	1.15
**26695*mutY* comp**	**J99-R3**	40	25	$2.28 \times 10^{+08}$
**26695*nth***	**J99-R3**	53	9	0.45
**26695*nucT***	**J99-R3**	29	4	0.24
**26695*recA***	**J99-R3**	0	0	1.0
**26695*recB***	**J99-R3**	51	7	0.22
**26695*recG***	**J99-R3**	63	8	0.18
**26695*recJ***	**J99-R3**	61	8	0.20
**26695*recJxseA***	**J99-R3**	55	8	0.26
**26695*recN***	**J99-R3**	59	2	0.20
**26695*recR***	**J99-R3**	69	6	0.11
**26695*ruvA***	**J99-R3**	41	5	0.18
**26695*ruvB***	**J99-R3**	1	0	0.55
**26695*ruvC***	**J99-R3**	41	7	0.43
**26695*ung***	**J99-R3**	56	7	0.18
**26695*xseA***	**J99-R3**	59	11	0.75
**26695*xth***	**J99-R3**	33	4	0.19

[1] Num clones = number of clones with DNA imports in *rpoB.*

[2] Approximated using the Bayesian Information Criterion (cf. Methods).
