# Supplementary material for: Mosaic DNA Imports with Interspersions of Recipient Sequence after Natural Transformation of Helicobacter pylori
Source: PLoS One. 2008 Nov 24;3(11):e3797. doi: 10.1371/journal.pone.0003797 (PMC2582958; doi:10.1371/journal.pone.0003797)
Supplement: Table S7 — Maximum likelihood estimation (MLE) of the mean length of ISR in the analyzed H. pylori wild type and mutant strains (0.02 MB PDF) [file pone.0003797.s007.pdf]

S. Kulick et al.

Supporting information

Table S7: Maximum likelihood estimation (MLE) of the mean length of ISR in the analyzed *H. pylori* wild type and mutant strains

Recipient strain	Donor DNA	Num clones[1]	Num ISR	MLE (bp)	Bayes Factor[2]
**26695**	**J99-R3**	95	9	39	
	**N6-R1**	26	3	121	1.20
**J99**	**26695-R1**	32	9	38	0.24
**N6**	**26695-R1**	25	6	110	1.62
	**J99-R3**	80	9	114	2.26
**26695*comB10***	**J99-R3**	0	0	-	-
**26695*comB10* EP**	**J99-R3**	25	13	112	2.47
**26695*magIII***	**J99-R3**	51	9	30	0.26
**26695*mfd***	**J99-R3**	29	3	206	9.88
**26695*mutS***	**J99-R3**	53	11	76	0.59
**26695*mutY***	**J99-R3**	43	10	22	0.39
**26695*mutY* comp**	**J99-R3**	40	52	116	3.16
**26695*nth***	**J99-R3**	53	10	87	0.88
**26695*nucT***	**J99-R3**	29	9	43	0.24
**26695*recA***	**J99-R3**	0	0	-	-
**26695*recB***	**J99-R3**	51	7	85	0.76
**26695*recG***	**J99-R3**	63	8	62	0.36
**26695*recJ***	**J99-R3**	61	8	78	0.61
**26695*recJxseA***	**J99-R3**	55	8	43	0.25
**26695*recN***	**J99-R3**	59	2	15	0.45
**26695*recR***	**J99-R3**	69	6	170	10.27
**26695*ruvA***	**J99-R3**	41	5	27	0.31
**26695*ruvB***	**J99-R3**	1	0	-	-
**26695*ruvC***	**J99-R3**	41	7	44	0.26
**26695*ung***	**J99-R3**	56	7	55	0.31
**26695*xseA***	**J99-R3**	59	11	78	0.65
**26695*xth***	**J99-R3**	33	4	113	1.39

[1] Num clones = number of clones with DNA imports in *rpoB.*

[2] Approximated using the Bayesian Information Criterion (cf. Methods).
