# Supplementary material for: Mosaic DNA Imports with Interspersions of Recipient Sequence after Natural Transformation of Helicobacter pylori
Source: PLoS One. 2008 Nov 24;3(11):e3797. doi: 10.1371/journal.pone.0003797 (PMC2582958; doi:10.1371/journal.pone.0003797)
Supplement: Supplementary References S1 — References for supplementary material (0.04 MB DOC) [file pone.0003797.s008.doc]

**S. Kulick et al.**

**Supporting information**

Reference List

S1. Hanahan D (1983) Studies on transformation of *Escherichia coli* with plasmids. *J Mol Biol* 166:557-580.

S2. Casadaban M, Cohen SN (1980) Analysis of gene control signals by DNA fusion and cloning in *E. coli*. *J Mol Biol* 138:179-207.

S3. Tomb J-F, *et al.* (1997) The complete genome sequence of the gastric pathogen *Helicobacter pylori*. *Nature* 388:539-547.

S4. Alm RA, *et al.* (1999) Genomic-sequence comparison of two unrelated isolates of the human gastric pathogen *Helicobacter pylori*. *Nature* 397:176-180.

S5. Ferrero RL, Cussac V, Courcoux P, Labigne A (1992) Construction of isogenic urease-negative mutants of *Helicobacter pylori* by allelic exchange. *J Bacteriol* 174:4212-4217.

S6. Kulick S, Moccia C, Kraft C, Suerbaum S (2008) The *Helicobacter pylori mutY* homologue HP0142 is an antimutator gene that prevents specific C to A transversions. *Arch Microbiol* 189:263-270.

S7. Ge Z, Hiratsuka K, Taylor DE (1995) Nucleotide sequence and mutational analysis indicate that two *Helicobacter pylori* genes encode a P-type ATPase and a cation-binding protein associated with copper transport. *Mol Microbiol* 15:97-106.

S8. Labigne-Roussel A, Courcoux P, Tompkins L (1988) Gene disruption and replacement as a feasible approach for mutagenesis of *Campylobacter jejuni*. *J Bacteriol* 170:1704-1708.

S9. Yanisch-Perron C, Vieira J, Messing J (1985) Improved M13 phage cloning vectors and host strains: nucleotide sequences of the M13mp18 and pUC19 vectors. *Gene* 33:103-119.

S10. Huang S, Kang J, Blaser MJ (2006) Antimutator role of the DNA glycosylase *mutY* gene in *Helicobacter pylori*. *J Bacteriol* 188:6224-6234.
