# Supplementary figures and images for: Mosaic DNA Imports with Interspersions of Recipient Sequence after Natural Transformation of Helicobacter pylori
Source: PLoS One. 2008 Nov 24;3(11):e3797. doi: 10.1371/journal.pone.0003797 (PMC2582958; doi:10.1371/journal.pone.0003797)

M    wt    1    2    3    +    -

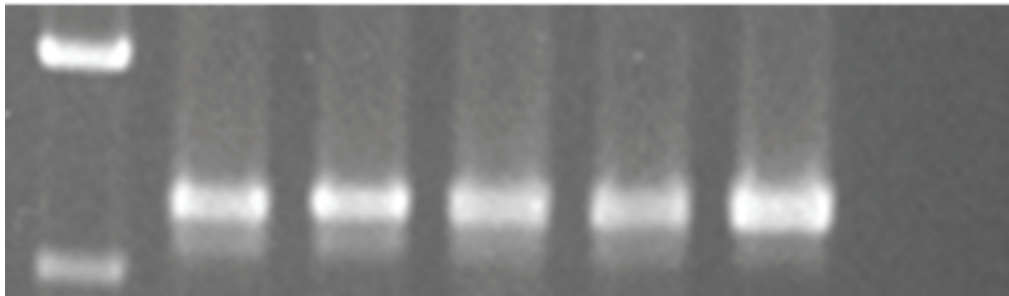

16 S

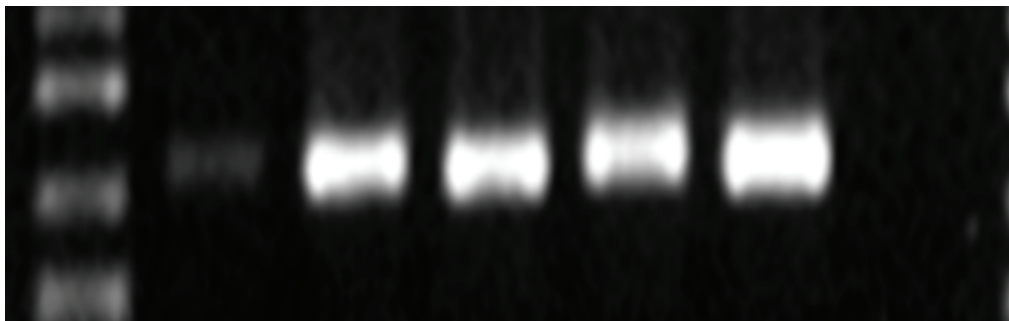

*mutY*

Supplement: Figure S1 — Semi-quantitative RT-PCR analyses of the abundance of mutY mRNA in the H. pylori strains 26695 and 26695mutYcomp. (0.12 MB PDF) [file pone.0003797.s009.pdf]
